# Supplementary material for: Dispensing of antibiotics without prescription and associated factors in drug retail outlets of Eritrea: A simulated client method
Source: PLoS One. 2020 Jan 24;15(1):e0228013. doi: 10.1371/journal.pone.0228013 (PMC6980490; doi:10.1371/journal.pone.0228013)
Supplement: S1 File — (PDF) [file pone.0228013.s001.pdf]

Data recording form for the sales of antibiotics without prescription

Section A: Background Information		
**No.**	**Indicators**	**Coding categories**
101	Code of the pharmacy outlet	☐☐
102	Type of the pharmacy outlet	Governmental chain pharmacy........................... 1<br>Private community pharmacy............................. 2<br>Private drug shop... 3
103	Zonal location	Maekel.. 1<br>Northern Red Sea .... 2<br>Debub .... 3<br>Anseba.. 4<br>Gash Barka .... 5<br>Southern Red sea... 6
104	Subzone name	______________________________
105	Sex of the professional	Male.. 1<br>Female 2
106	Estimated age group (in years)	Below 30 years .... 1<br>30 – 50 years .... 2<br>Above 50 years... 3
107	Does the professional license photo matched with the pharmacy attendant?	Yes.. 1<br>No .... 2<br>Not sure .... 3<br>Not found... 4
108	Actual age (in years)	☐☐
109	Title of the professional	Pharmacist .... 1<br>Pharmacy technician .... 2<br>Nursing Degree .... 3<br>Nursing Diploma .... 4<br>Health assistant.. 5<br>Other *(please specify)*______________________ 6
110	Year of experience	☐☐

Section B: Information related to Antibiotic dispensing			
**No.**	**Indicators**	**Coding categories**	**Skip**
201	Type of the clinical scenario	Acute watery diarrhea .... 1<br>Uncomplicated UTI 2	
202	Was an antibiotic dispensed?	Yes .... 1<br>No 2	2 → **205**
203	At which level of demand was the antibiotic dispensed?	Demand 1 .... 1<br>Demand 2 .... 2<br>Demand 3 3	
204	Name of the antibiotic(s) dispensed<br>***(Multiple answers are possible)***	Ciprofloxacin .... A<br>Co-trimoxazole .... B<br>Metronidazole .... C<br>Amoxicillin .... D<br>Doxycycline .... E<br>Other *(please specify)* _________ X	A–X → **End of entry**
205	Reason for not selling an antibiotic<br>***(Multiple answers are possible)***	Administrative issues .... A<br>Referral to health facility .... B<br>Illness doesn't require an antibiotic C<br>Fear of complications .... D<br>Unavailability of an antibiotic ...... E<br>Other *(please specify)* _________ X	
206	Name of the alternative medication dispensed in place of an antibiotic, if any	______________________________	

Data Collector Code [ ][ ] **Date** ____________________________

Comment Section
